# Changes in Regenerative Capacity through Lifespan

**DOI:** 10.3390/ijms161025392

**Published:** 2015-10-23

**Authors:** Maximina H. Yun

**Affiliations:** Institute of Structural and Molecular Biology, Division of Biosciences, University College London, Gower Street, London WC1E 6BT, UK; E-Mail: maximina.yun@ucl.ac.uk; Tel.: +44-(0)-207-679-4493

**Keywords:** aging, regeneration, senescence, stem cells, reprogramming, newt, axolotl, zebrafish, planaria

## Abstract

Most organisms experience changes in regenerative abilities through their lifespan. During aging, numerous tissues exhibit a progressive decline in homeostasis and regeneration that results in tissue degeneration, malfunction and pathology. The mechanisms responsible for this decay are both cell intrinsic, such as cellular senescence, as well as cell-extrinsic, such as changes in the regenerative environment. Understanding how these mechanisms impact on regenerative processes is essential to devise therapeutic approaches to improve tissue regeneration and extend healthspan. This review offers an overview of how regenerative abilities change through lifespan in various organisms, the factors that underlie such changes and the avenues for therapeutic intervention. It focuses on established models of mammalian regeneration as well as on models in which regenerative abilities do not decline with age, as these can deliver valuable insights for our understanding of the interplay between regeneration and aging.

## 1. Introduction

From the onset of development until the end of their lifespan, most organisms experience a progressive decline in their regenerative abilities. From a biological perspective, regeneration can be subdivided into the ability to replace lost or damaged cells, which includes tissue turnover and limited injury responses found in the majority of organisms including mammals, and the ability to regenerate complex structures, which is mostly absent in mammals but finds expression in a number of other animals. During aging, mammals exhibit changes in their ability to regenerate vital biological structures such as the vascular, nervous, muscular, haematopoietic and skeletal systems as well as many organs and cell types, which correlate with the overall organismal decay. Although metazoan species exhibit a diverse range of lifespans, it is notable that in most organisms studied so far there is a strong association between the decline in regenerative capacity and the aging process. Indeed, it has been proposed that aging results from the inability to maintain proper tissue structure and function due to insufficiencies in regenerative capacity [1]. Hence, regeneration and aging could represent two sides of the same coin. This idea is supported by the existence of organisms with extreme regenerative capacities, such as planarians and salamanders, which exhibit negligible signs of aging, as indicated by the lack of measurable functional declines with age [2].

The principles that underlie the decline in regenerative abilities through lifespan are currently being unravelled. However, it is already clear that both cell-intrinsic (such as cellular senescence) as well as cell-extrinsic factors (such as alterations in the regenerative environment) play significant roles. Notably, these factors show extensive overlap with those known to underlie the aging process [3], highlighting the interconnection between aging and regeneration and stressing that therapeutic approaches designed towards enhancement of regenerative abilities could also result in considerable health/lifespan improvements.

This review discusses the nature of the changes in regenerative abilities that take place through lifespan and across phylogeny, the factors which underpin such changes and the avenues for therapeutic interventions which leverage off this body of research. A particular emphasis is placed on knowledge derived from the classic regeneration models, organisms capable of extensive regeneration of complex structures in which age related declines in regenerative abilities are not observed, as this can shed light on important mechanisms with potential therapeutic application.

## 2. Changes in Regenerative Ability through Phylogeny, Ontogeny and Aging

**Figure 1:**
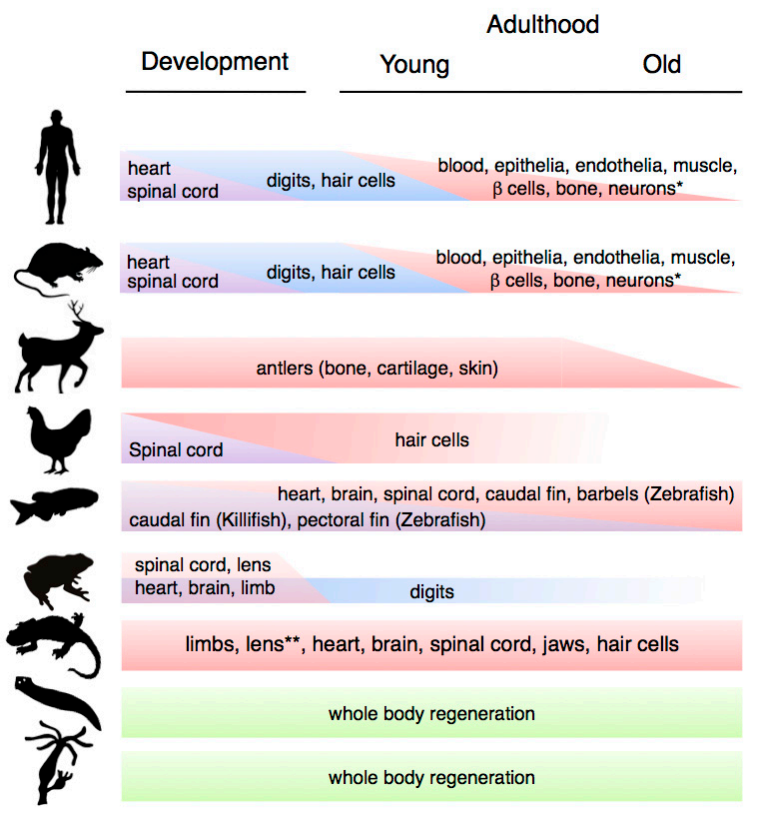
Variation in regenerative capacity through phylogeny, ontogeny and aging. * Note that the ability to regenerate the indicated systems is present in most other animal groups; ** Lens regeneration is observed throughout lifespan in newts, but it can only occur during a limited developmental window in axolotls.

The ability to regenerate tissues, organs and body structures varies drastically across the animal kingdom (Figure 1). This variation ranges from the ability to execute whole body regeneration found in Hydra and freshwater planarians, through the extensive abilities to regrow complex structures found in vertebrates such as teleost fishes (e.g., zebrafish) and salamanders (e.g., newts and axolotls), to the more limited regeneration capacities found in mammals. Furthermore, within a particular organism, these abilities may vary according to the nature of the tissues and the age of the organism. Studies in several regeneration systems have uncovered two important overall trends. First, tissue regeneration tends to be high during early life stages, and various regenerative time-windows have been defined through ontogenesis [4]. Second, there is a progressive decline in regenerative abilities during aging. These tendencies are valid for many organisms, including mammals. However, there are interesting exceptions, which include the classic models of regeneration such as planarians, zebrafish and salamanders.

Schematic depiction of changes in regenerative capacity at different life stages (development, early and late adulthood) in representative organisms. From top to bottom: mammals (human, mouse, deer), birds (chicken), fish (zebrafish/killifish), amphibians (frog, salamander (newts and axolotls)), Platyhelminthes (planarian flatworm) and cnidarians (hydra). Whereas a progressive decline in regenerative abilities is characteristic of mammalian aging, classical regeneration models such as hydra, planarians, zebrafish and salamanders exhibit extensive and time resistant abilities to regenerate complex structures.

### 2.1. Variations during Development

Ontogenetic changes in regenerative capacity occur in many physiological systems, including heart, spinal cord, digit tips and hair cells (Figure 1). For example, mammals have the ability to regenerate their hearts until shortly after birth, when this ability is lost [5]. In addition, they are only able to regenerate their spinal cords during early development (and then only partially), in common with other organisms such as birds and frogs [6].

Among the cell types whose regeneration is affected by the developmental stage, auditory hair cells constitute a well-characterised example. In contrast to salamanders, birds and fish, which can regenerate their hair cells through adulthood [7], in mammals this is only possible during development [8]. Inter and intra-species comparative experiments suggest that fluctuations in key developmental regulators such as retinoic acid and growth factors underlie changes in hair cell regenerative capacity [8].

Ontogenetic changes also underlie the ability to regenerate digit tips in mice and humans, as this ability drastically decreases after development. Foetal digit tip regeneration is rapid and completed by birth, whereas neonatal digit tip regrowth takes considerably longer and occurs with imperfections [9,10]. In a more striking example, frogs are able to regenerate full limbs prior to metamorphosis, however after this process they are only capable of regenerating their digits. Indeed, post-metamorphic changes in frogs are dramatic, affecting their ability to regenerate not only limbs and spinal cord but also their heart, skin and lens [4]. Interestingly, extending the time a frog spends as a larva leads to increases in its ability to regenerate the spinal cord [11]. This suggests that the hormonal or structural alterations brought about by metamorphosis could impact on the ability to regenerate certain tissues. However, there are other examples among amphibians that do not seem to be related to metamorphosis-induced changes, such as the case of the axolotl lens, which can only be regenerated for a period of two weeks after hatching [12].

Although the factors that determine these ontogenetic changes remain unknown, it is possible that the growth stimuli and high cellular plasticity levels associated with developmental processes contributes to the generation and instruction of regenerative progenitors, creating a permissive regenerative environment during development. As the structure, physiology and environmental exposure of an organism changes from development to adulthood, it is likely that changes in regenerative capacity that occur late during lifespan are driven by different factors than those operating during development.

### 2.2. Variations during Aging

Aging is associated with defects in tissue regeneration and repair that lead to cell loss and compromise of tissue homeostasis, structure and function. This is particularly well documented in mammals, whose regenerative functions predominantly rely on tissue-specific stem cells (cells with the ability to self-renew and produce daughter cells that differentiate into particular cell types), and in some cases on restricted progenitor cells (cells which can undergo cell division despite a degree of maturation). With age, both stem and progenitor cells undergo a series of alterations including loss of self-renewal capacities, altered proliferative activity, declines in functionality and potency. These changes have been shown to contribute to the degeneration and dysfunction of a number of tissues and systems including blood, muscle, bone, cartilage, the central nervous system (CNS), organs such as the pancreas, and most epithelia and endothelia [13,14].

The regenerative capacity in the skeletal muscle system experiences a marked decline with age in many organisms, as reflected by a decrease in the generation of myofibres and an increase in fibrotic tissue upon muscle injury [13,15]. In humans, this is an underlying cause of sarcopenia, the loss of muscle mass that accompanies aging. The decline in muscle regenerative potential is largely attributed to changes in satellite cells, the muscle stem cells, which undergo age-related declines in proliferative and myogenic capacities. Indeed, satellite cell numbers decline gradually in mammalian muscles with advancing age [16]. As discussed in the next section, these changes seem to be the result of both cell-intrinsic as well as extrinsic alterations such as aging of the muscle environment.

In mammalian species, age-related declines in immune responses, increase in leukaemia incidence and normocytic anaemia are associated with a decline in regenerative potential of haematopoietic stem cells (HSC) [17]. Age-related changes in HSC include decreases in functionality, changes in cell cycle kinetics and skewed differentiation towards myeloid lineages [18,19,20,21].

Age-specific changes have also been reported for mesenchymal stem cells (MSC), stromal cells that can differentiate into multiple cell types such as osteblasts, condrocytes and adipocytes. Alterations include a loss in chondrogenic potential leading to impaired chondrocyte formation, which results in decreased cartilage repair in aged mammals [22]. Furthermore, studies in human-derived bone marrow MSC revealed age-dependent decreases in their capacity to differentiate to osteoblasts, which are related to increases in the level of MSC apoptosis and senescence upon aging [23,24]. Together, these alterations contribute to conditions such as osteoporosis and reduced bone repair capacity that are characteristic of human aging [23].

In mammals, the extent of adult neurogenesis also declines with age. In humans and mice, adult neurogenesis occurs in the hippocampus and the olfactory bulb [25]. *De novo* neurogenesis takes place from neural stem cells (NSC), and these have been shown to decline with age, resulting in significant decreases in regenerated neurons and hence functional impairment [13]. Factors that underlie such defects include reduced stem cell proliferation and increased apoptosis in the regenerated neuronal progeny [26]. Furthermore, age also affects regeneration of peripheral nerves, as exemplified by the loss of taste bud regeneration in old mice as a result of impaired recovery from gustatory nerve injury [27]. This is an interesting example of nerve dependence in regeneration, an important phenomenon which should also be considered in the context of changes in regenerative abilities upon aging (see discussion in the Section 3.7).

Pancreatic β cells, the key centres of insulin production, storage and release, constitute a clear example of age-dependent alterations in regenerative capacity. In both humans and mice, pancreatic β cells increase in number (upon physiological or regenerative stimuli) largely through compensatory proliferation [28], although transdifferentiation from pancreatic α and δ cells has also been reported in cases of extensive β cell loss in mice [29,30]. With age, β cell turnover capacities decline drastically [31]. This has been associated with changes in key cell-cycle regulators, such as the epigenetic derepression of p16^ink4a^, as well as the activation of p38 kinase [32,33]. Interestingly, recent studies in mice suggest that, concomitantly with a decline in replicative potential, there is an improvement of β cell functions such as insulin-secretion with aging, highlighting that aging does not always result in declines of cellular function [34].

As mentioned, aging also affects the regenerative capacity of progenitor cells. Age-related changes in endothelial progenitor cells (EPC), circulating progenitors with an endothelial phenotype that contribute to the regeneration and repair of vessel walls, have been reported. Although there is no change in the number of EPC with age, deficits in cell function are apparent during aging [35]. In mice, this has been associated with the development of atherosclerosis, a common disease of old age [36].

### 2.3. The Exceptions to the Rule

**Figure 2:**
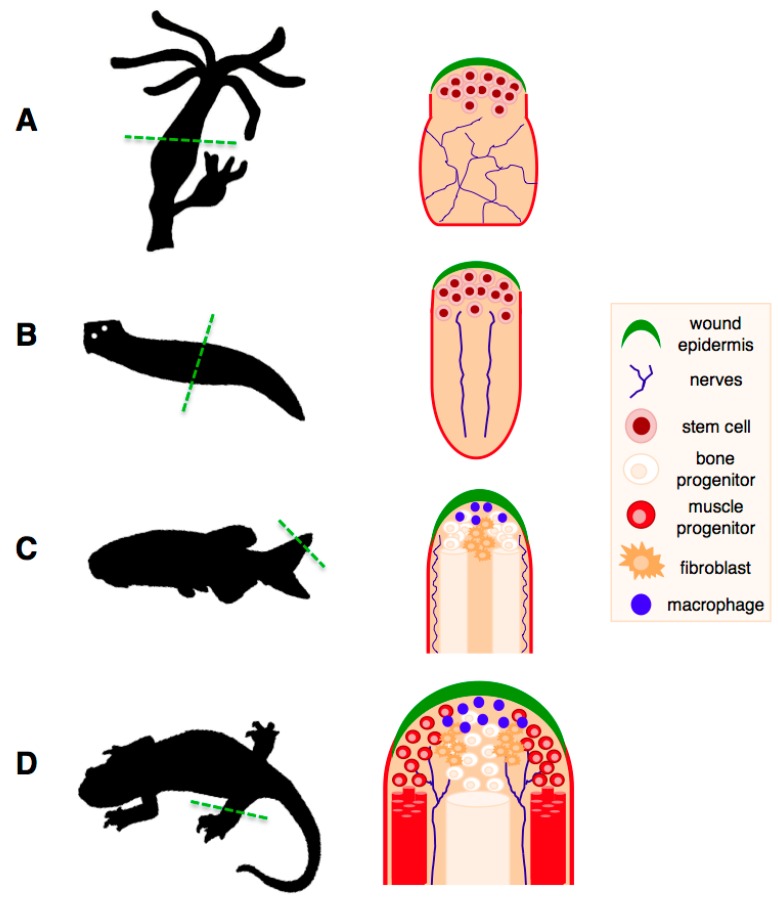
Regeneration of complex structures in classic regeneration models. (**A**) Regeneration of a hydra polyp following amputation across the body stalk. Regeneration takes place through mobilisation and activation of multipotent endodermal and ectodermal stem cell populations; (**B**) Regeneration of a planarian flatworm following bisection. This process takes place through recruitment of pluripotent stem cells, termed “neoblasts”, which are present throughout the animal and carry out tissue maintenance functions. A single clonogenic neoblast is capable of regenerating an entire organism; (**C**) Regeneration of the zebrafish fin. Upon amputation of the fin, differentiated cells at the amputation plane undergo dedifferentiation and proliferate to form a pool of progenitors called a blastema, which will then undergo growth and redifferentiation into the new fin tissues; (**D**) Salamander limb regeneration depends, as in the zebrafish case, on the dedifferentiation of mature cells from the tissues at the amputation plane. Unlike the zebrafish fin, which grows continuously, salamander regeneration takes place in the context of mature adult tissues. Both in zebrafish and salamanders, the dedifferentiation process generates progenitors of limited potential, which can only regenerate their tissues of origin. The wound epithelium, nerve supply and macrophages are critical components of the regenerating niche, without which regeneration cannot proceed. Adapted from Brockes *et al.* [48].

While the aforementioned observations are true for most organisms studied so far, this is not valid for a number of organisms, the classic regeneration models, such as hydra, planarians, zebrafish and salamanders (Figure 2). Not only do they exhibit the most extensive regenerative abilities in the natural world, but also these abilities remain intact throughout their lifespan.

Two remarkable organisms, the freshwater cnidarian hydra and the planarian flatworm represent the most extreme case, as they are able to regenerate whole bodies from a single fragment [37,38,39]. Interestingly, this type of regeneration is based on stem cells with a high level of plasticity, and constitutes the basis for the asexual reproduction strategy used by these organisms. In the case of planarians, their remarkable regenerative abilities led to the conclusion that they may “almost be called immortal under the edge of the knife” [40]. Studies on these models raise the question of what factors underlie their impressive regenerative capabilities and to what extent this knowledge could be translated to mammals.

Perhaps even more remarkably, examples of high and time-resistant regenerative abilities are found among vertebrates. This includes the zebrafish, which is able to regenerate complex structures such as parts of its heart and brain, barbels, fins and spinal cord [41,42]. Notably, detailed studies in young and old individuals have revealed that the ability to regenerate the caudal fin, the barbels and the heart does not decrease with age [43,44] or repetitive regeneration cycles [44,45,46] although age and sex-related differences do affect regeneration of the pectoral fin [47].

Extensive regenerative abilities are also found in the salamanders, such as newts and axolotls. Among vertebrates, these organisms are considered the champions of regeneration, as they are able to regrow a wide range of structures including parts of their heart and brain, jaws, entire tails (including the spinal cord), lens and hair cells, and they are the only vertebrate able to regenerate full limbs in the adult stage [48,49]. Notably, whereas regeneration in hydra, planarians and zebrafish takes place in organisms that are constantly growing or undergoing high turnover, which could be an important precondition of their regenerative abilities [42], salamander regeneration operates in the context of an organism that has reached its maturation stage. In these organisms, regeneration of complex structures takes place through reprogramming of differentiated cells, proliferation, and subsequent redifferentiation of adult tissues [48,50], although transdifferentiation and stem-cell based regeneration are also used in certain contexts [51,52]. Importantly, their ability to regenerate their limbs or spinal cords does not decline with repetitive amputations [53], suggesting that these organisms have an unlimited regenerative potential for regenerating complex structures. Indeed, these observations are supported by a recent study on lens regeneration, which suggests that regenerative capacity in newts is not altered by repeated regeneration and aging [51].

Together, these organisms demonstrate that attaining perfect regeneration even during old age is possible, although the mechanisms that underlie such unlimited regenerative capacities remain unknown. Recent technological advances (e.g., transgenesis, development of RNA sequencing technologies) have transformed many of these organisms into valuable, tractable models in which to study the factors and mechanisms that impact on the decay or resistance of regenerative capacity upon aging.

## 3. Factors that Affect Regenerative Capacity during Aging

**Figure 3:**
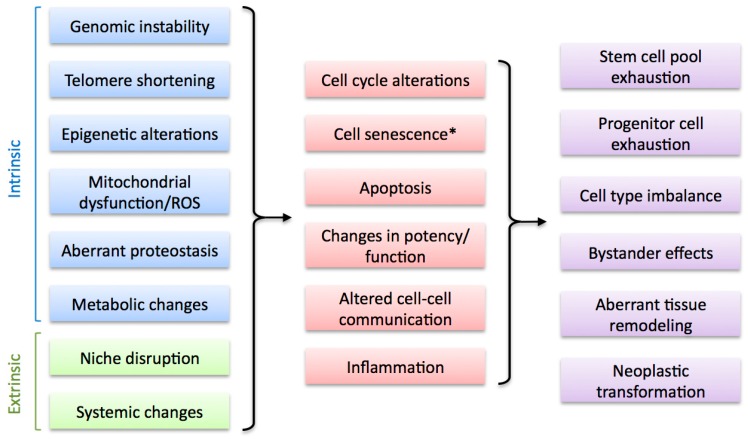
Factors that alter regenerative capacity upon aging. Multiple cell-intrinsic and cell-extrinsic factors are associated with the decline of regenerative capacity during aging (**left**). These impact on regenerative cell types by altering key cellular processes (**middle**), leading to various negative outcomes that result in regeneration impairment (**right**). * Cell senescence can be considered an intrinsic and, potentially, extrinsic factor (see Section 4).

A number of cellular and molecular mechanisms have been associated with the decline in regenerative abilities observed during aging in humans and various model organisms (Figure 3). Unsurprisingly, most of these mechanisms have also been shown to be important contributors to the aging process and form part of the recently defined Hallmarks of Aging [3]. These include intrinsic factors such as genomic instability (including telomere attrition), mitochondrial dysfunction, epigenetic changes, loss of proteostasis and metabolic alterations, as well as cell extrinsic factors such as disruption of the regeneration niche and alterations in systemic signals. Though most of these factors can contribute to age-related impairment in regenerative capacity, a consensus on their relative importance in this process is currently lacking. Furthermore, emerging evidence suggests a high degree of interconnectivity between them, stressing the importance of identifying the common denominators which will help us define the underlying causes of the decline in regenerative abilities upon aging and derive successful therapeutic strategies.

### 3.1. Genomic Instability

In the majority of cases, the process of regeneration relies on cells with long-term capacity for self-renewal. While this characteristic is key for their ability to promote regenerative processes through lifespan, it also increases the possibility of acquiring DNA damage as a result of replicative stress, including from telomere erosion (discussed below) as well as internal and external mutagens (e.g., reactive oxygen species (ROS) and UV light exposure respectively). Indeed, various studies have shown a marked accumulation of DNA damage with age in mammalian stem cells [21,54,55,56,57]. This poses important challenges, as these genotoxic lesions could impact on gene function, leading to alterations in differentiation and self-renewal capacities, and promote neoplastic transformations resulting in increased cancer risk. In addition, somatic mutations could affect cells within the regenerative niche, leading to functional impairments that affect the regeneration outcome.

To counteract such threats the cells rely on a sophisticated DNA damage response machinery, which allows detection and repair of various types of DNA damage through mechanisms such as mismatch repair, nucleotide excision repair, non-homologous or microhomology-mediated end joining, and homologous recombination. This network also includes checkpoint mechanisms that promote transient cell cycle halts for the repair of genotoxic lesions and ensures that such repair has been carried out. When the damage is too extensive or cannot be repaired, this machinery leads to the elimination via apoptosis, or permanent cell cycle arrest via cellular senescence, of the compromised cells. In the context of regeneration, this can result in depletion of stem/progenitor cell pools [58], loss of stem cell quiescence [59], premature differentiation [60] and, when the checkpoint mechanisms fail and the DNA damage remains, neoplastic transformation.

The importance of the maintenance of genomic stability for regenerative processes is further highlighted by the fact that many mutations that disrupt the ability of cells to detect and repair DNA damage result in impaired regenerative capacity in various tissues. These include mutations in the DNA damage sensor ATM and in the RecQ DNA helicases, which lead to the Ataxia Telangiectasia and the Werner, Blood and Rothmund-Thompson progeroid syndromes respectively (human diseases that recapitulate some aspects of chronological aging) [61]. In particular, mesenchymal stem cells reprogrammed from Werner syndrome patients exhibit premature cellular senescence [62]. Additionally, DNA repair defects such as those found in cells derived from Fanconi anemia patients (which have defects in crosslink repair and homologous recombination proteins) can predispose cells to catastrophe during proliferation bursts, as shown in the case of haematopoietic stem cells which undergo cell death following physiological challenges such as substantial blood loss.

In mice, genetic disruption of ATM or another DNA damage sensor, ATR, causes significant age-dependent perturbation in stem cell compartments. Furthermore, stem cells from mouse models deficient in DNA repair pathways such as non-homologous end joining, crosslink and nucleotide excision repair show diminished self-renewal, increased apoptosis and functional exhaustion with age [21,58]. In addition, defective DNA repair mechanisms have been shown to sensitise hair-follicle melanocyte stem cells to undergo premature differentiation, leading to stem cell pool depletion and accelerated hair greying [63].

The extent to which these repair mechanisms can affect regenerative progenitors depends on the nature of the mechanism and cell-cycle status of the cell. For example, in many organisms the maintenance of pools of quiescent cells is critical for regenerative capacity. However, this requirement ironically contributes to the damage or depletion of these cells as the quiescent state, with its single set of chromatids, precludes the use of accurate forms of DNA repair (such as homologous recombination) and promotes error-prone non-homologous end joining [64]. In the case of aging hematopoietic stem cells, this has recently been shown to result in a quiescent-dependent and marked accumulation of DNA damage coinciding with a broad attenuation of DNA repair and response pathways [65]. Therefore, the interplay between cell cycle status and DNA repair pathways can impact the extent of increased genomic instability with age, which in turn has consequences for regenerative capacity.

Recently, various studies have found age-dependent abnormalities in expression of DNA repair genes [65,66], which correlate with defects in stem cell functionality. Interestingly, recent gene expression analysis during lens regeneration in adult newts, a process unaffected by aging, revealed an upregulation of DNA repair genes during regeneration [14,67]. This could represent a strategy to minimise genomic instability, promoting accurate regeneration even at an advanced age, something which should be explored in further studies.

In addition to age-related changes in the expression or activity of DNA repair genes, the expression of DNA replication genes can also change during aging, leading to further genomic compromise derived from replicative stress [54]. A comparison between young and old HSCs recently uncovered a decrease in expression of MCM4 and MCM6 (part of a helicase complex essential for replication) upon aging, coinciding with persistent DNA damage. Such damage was particularly apparent in ribosomal DNA genes, known to be sensitive to replicative stress as they pose replication challenges. This led to a decreased expression of ribosomal components resulting in impaired cellular functions and regenerative capacity, highlighting the role of replicative stress in age-related declines.

Although the exact mechanisms by which genomic instability affect regenerative capacities are still being elucidated, extensive evidence points at the role of the DNA damage checkpoint network and in particular its key effectors, the gatekeeping tumour-suppressors p53, p16^ink4a^ and p19/p14^arf^. They function by promoting cell cycle arrest, apoptosis and senescence. Therefore, while constituting powerful antitumourigenic mechanisms, they tend to negatively affect stem/progenitor cell function. As with DNA damage, their expression or function increases with age [66,68,69,70,71]. p16^ink4a^ is an essential effector of cellular senescence which has been linked to the promotion of aging phenotypes including the decline in regenerative capacity in a variety of cell types such as muscle, brain and bone marrow (see discussion on the role of senescence below). In contrast, the functions of p19^arf^ on regenerative processes are less well-defined; however, it is thought to act by regulating the tumour suppressor p53.

Named “the guardian of the genome” [72], p53 is activated by DNA damage including telomere erosion (see below), reactive oxygen species and oncogene activation, leading to a transient cell cycle arrest, apoptosis or senescence depending on the severity of the stress incurred [73]. Many reports suggest that an increase in p53 expression or activity levels can lead to aging and reduced lifespan, and lead to stem cell death in response to telomere attrition [74,75,76]. Mice carrying a hyperactive mutant form of p53 exhibit high levels of cellular senescence in various tissues and defects in the HSC compartment [77,78]. In line with these observations, the elimination of p53 in mice that exhibit short telomeres can rescue their defects in regenerative responses and wound healing (see below). Furthermore, a transient reduction in p53 activity has been recently shown to be required for the regeneration of full limbs in salamanders, by promoting the dedifferentiation of adult cells to generate progenitors for regeneration [50]. Together, these findings show that p53 is important for the regulation of regenerative potential across various regeneration modalities (natural reprogramming and stem cell-based regeneration), and suggest that increases in p53 activation, such as that frequently found in aged tissues, are detrimental for regeneration. However, there is also evidence suggesting that p53 can be beneficial to regenerative processes, including observations in mice carrying an additional copy of the intact p53 locus. Surprisingly, these mice show a delayed onset of aging, extended lifespan and normal regeneration and wound healing phenotypes [79,80]. These seemingly conflicting results are likely to be related to the fact that the functions of p53 are highly dependent on its dose, activation status and kinetics, something that extends to other important gatekeepers and should be considered when interpreting findings and devising strategies to promote regeneration. Lastly, it should be noted that even though p53 inactivation could deliver improvements in regenerative capacity, these are outweighed by the associated risks in cancer development. Further studies focussing on the control of particular molecular functions by this important tumour suppressor, as well as studies in organisms such as salamanders, which can successfully modulate p53 to increase their regenerative potential without increasing cancer risk, will shed light on the mechanisms by which p53 impacts on regeneration and open avenues for therapeutic exploration.

### 3.2. Telomere Shortening

A particular type of genomic instability that has been linked to the decline in regenerative capacities upon aging is telomere attrition. Telomere shortening, initially associated with the triggering of replicative senescence in proliferating cells *in vitro* [81], has been also observed *in vivo* during normal mammalian aging [82], in a number of regenerative cell types such as hair follicle [83] and hematopoietic stem cells [84].

Telomeres are tracts of multiple nucleotide repeats, highly conserved through evolution, and located at chromosome ends [85,86]. They associate with a number of proteins, such as the shelterin complex [87], forming a cap that prevents the activation of the DNA damage response, which could otherwise lead to widespread genomic instability driven by chromosome breakage-fusion events [88]. Telomere length is commonly maintained by the cellular retrotranscriptase telomerase, an RNA-dependent DNA polymerase capable of replicating telomeres and thus overcoming the end-replication problem posed by linear telomeric sequences [89]. Whilst high in cells of the germ line, largely due to their elevated levels of telomerase, telomere length decreases progressively with each cell cycle round in somatic cells, acting as a bona fide molecular clock. When telomeres become too short, they elicit a strong DNA damage response that results in apoptosis or cellular senescence. Hence, telomere attrition poses a serious mechanistic problem for highly proliferative cells including regenerative and cancer cell types. For this reason, stem and progenitor cells, with their need for extensive replication, are particularly vulnerable to telomere shortening. Indeed, in humans, telomere attrition is associated with a number of disorders that lead to loss of regenerative capacity, including dyskeratosis congenita, a progeroid syndrome that originates from a mutation in a key component of the telomerase complex, DKC1, and results in the inability to reconstitute the bone marrow [90].

Observations in mouse models further support the role of telomeres as important factors underlying the decline in regenerative capacity with aging. Genetic disruption of the gene encoding the RNA component of telomerase (*Tertc*) leads a severe degenerative phenotype characterised by stem cell depletion, impaired responses to tissue injury and functional damage in a variety of tissues [91]. Defects in proliferation are found in epidermal stem cells, loss of self-renewal and differentiation impairments are observed in neural stem cells, and increases in apoptosis lead to depletion of intestinal stem cells [92,93,94]. As suggested, telomere shortening could provide unrepaired DNA damage signals that result in the activation of gatekeepers leading to cell cycle arrest, apoptosis or senescence. In agreement with this, the phenotype of mice with short telomeres (*Tertc*^−/−^) can be overcome by genetic disruption of p53. This results in a significant increase in proliferative abilities, a decrease in apoptosis, and improved wound healing responses and functionality in various tissues such as intestines, skin and the haematopoietic system [95,96]. Nevertheless, such mice exhibited an increased tumour incidence. In stark contrast, the cross of wild type and late generation *Tertc*^−/−^ mice results in healthy offspring, suggesting that both the telomere-dependent chromosomal instability and premature aging phenotypes can be reversed. Indeed, recent studies show that telomerase reactivation can reverse tissue degeneration in mice with shortened telomeres [97], and improve wound healing as well as regenerative responses in the mouse heart [98]. In this context, an important question is whether an increase in telomerase activity would, like p53 deletion, promote regenerative processes at the expense of cancer promotion. Notably, elevated telomerase activity has been reported in highly regenerative vertebrate species such as sea urchins and planarians, species in which tumour formation is rarely observed [99,100], suggesting that active telomere extension could contribute to extend regenerative capacities upon aging, and do so in a tumour-free manner. Moreover, recent studies in mice suggest that constitutive telomerase expression does not increase cancer risk [101]. Together, these observations have highlighted the relevance of telomere maintenance for the preservation of regenerative capacities during aging, and reignited interest in the development of therapeutic strategies designed to restore telomere integrity and hence promote regenerative capacity upon aging.

### 3.3. Mitochondrial Dysfunction and Oxidative Damage

Increases in mitochondrial dysfunction and bioenergetic deficiencies are characteristic of the aging process. Several studies have highlighted a connection between mitochondrial dysfunction and age-related declines in mammalian regeneration, however the identification of the relevant mitochondrial mechanisms has been difficult due to the multifunctional nature of these organelles: they constitute a vital metabolic centre, they serve as the primary sources of ROS, and play key roles in apoptosis.

Early studies led to the formulation of the mitochondrial free radical theory of aging, whereby the dysfunction of the respiratory chain results in increase production of ROS, which causes further deterioration, damage of the mitochondrial genome, and global cellular impairment [102]. Even though numerous studies support a role for ROS accumulation in age-related deterioration, recent observations have challenged this theory. For example, increasing mitochondrial ROS or oxidative damage does not result in accelerated aging in mice [103], and long-lived mammals such as the naked-mole rat exhibit high levels of ROS and oxidative damage [104]. Furthermore, ROS have recently emerged as important signals for regenerative responses in highly regenerative organisms, such as hydra, drosophila, planarians, zebrafish and amphibians [105,106]. It is now clear that they serve as one of the earliest signals for the recruitment of the immune components to sites of injury, enabling wound detection and subsequent regeneration. Moreover, they have been shown to modulate key regenerative pathways, promote proliferation of progenitors for the regenerate (in many cases compensatory, following ROS-mediated cell death induction) and even contribute to differentiation events [107,108,109,110,111]. In mammals, ROS can recruit stem cells to proliferate or differentiate [112]. Thus, rather than hindering regenerative responses, the right levels of ROS may in fact be critical for their success.

In addition, subsequent work suggests that mitochondrial dysfunction does affect aging, but that the accumulation of mutations in the mitochondrial genome constitutes the critical mechanism. Mice carrying a mutation in the proofreading domain of the mitochondrial polymerase, polymerase g [113], accumulate mutations in their mitochondrial DNA during development and this causes early-onset stem cell dysfunction and a premature aging phenotype [114]. Interestingly, the effects on stem cell populations vary according to the tissue. In the haematopoietic system, blocks in differentiation and/or disappearance of the haematopoietic progenitors are observed, leading to anaemia and lymphopenia, but there is no impact on HSC [115]. On the other hand, there is an impact on neural stem cells, as reflected by loss of quiescence and decrease in self-renewal capacities [116]. Together, these observations suggest that mitochondrial dysfunction could potentially accelerate aging and impact regenerative capacity but through a mechanism related to mitochondrial DNA mutation rather than ROS.

However, these findings do not exclude that ROS may still play a role downstream of the mitochondrial mutations in certain contexts as, notably, defects in neural stem cells and haematopoietic progenitors can be rescued by treatment with the antioxidant *N*-acetyl-l-cysteine [116]. It is possible that mitochondrial mutations may eventually lead to defects in the electron transport chain that could increase ROS levels in the affected cells. ROS could then trigger stress responses such as apoptosis, senescence and functional changes leading to stem cell dysfunction. In agreement with this, accumulation of oxidative damage has been observed in mice and human HSC after serial transplantations, which leads to expression of cell cycle inhibitors and premature senescence, causing loss of stem cell function [117].

Alternatively, mitochondrial mutations could impact on regenerative abilities independently of changes in the oxidative environment, through affecting proteins involved in apoptotic or intracellular signalling in stem cells or their niches [3,118,119]. In addition, a recent study suggests a role for the mitochondrial unfolded protein response (mtUPR) in stem cell aging (see below). It is clear that many questions abound on the mechanisms by which mitochondrial dysfunction impact on regenerative abilities, their hierarchical structure and interconnectivity, which future research should addressed.

### 3.4. Epigenetic Modifications

Changes in the epigenome such as DNA methylation and post-translational histone modifications are frequent during aging and have recently emerged as potentially critical factors contributing to age-associated decline in regenerative abilities [120]. The susceptibility of epigenetic modifications to intrinsic as well as extrinsic signals indicates that they could act as a point of convergence for many processes that impair regeneration upon aging. Furthermore, the reversible nature of these changes has heightened the interest in this area from a therapeutic perspective.

Large-scale changes in DNA methylation during aging have been reported for a number of species including human, mouse and rat [121,122,123]. While the observed tendency is a global decrease in methylation upon aging, the opposite is seen in certain genomic regions such as CpG islands, which exhibit hypermethylation with time [121]. This has also been observed in MSC following replicative senescence [124]. Some of these hypermethylated sequences are found within gene promoters, leading to alterations in gene expression that result in phenotypic changes and could impact on the regenerative capacity of a cell. Indeed, as mouse HSC age they accumulate changes in DNA methylation which could affect their self-renewal and differentiation abilities [65,125]. In support of this idea, modulators of DNA methylation are required for the function of many adult stem cells [126,127,128,129,130]. Furthermore, epigenetic regulation plays central roles during regeneration of complex structures, as demonstrated by experiments in the zebrafish fin, where loss of H3K27me3 is required for the initiation of the regenerative response [131], and in the cricket leg, where regulation of methylation/demethylation cycles are involved in leg patterning [132].

Age-related alterations in histone modifications, in particular methylation and acetylation, have been found in many contexts, from flies to humans. Their importance for regenerative processes is highlighted by studies modulating members of the sirtuin family of histone deacetylases, which have evolutionarily conserved roles in lifespan extension. For example, the expression of the mitochondrial sirtuin SIRT3 is downregulated during HSC aging, coinciding with defects in regenerative capacities. Strikingly, SIRT3 upregulation in these cells can revert the age-associated decline [133]. In addition, SIRT6 has been shown to confer protection from telomere and replicative stresses, preventing decreases in regenerative capacity in endothelial cells [134], while SIRT1 has been shown to prevent the onset of cellular senescence in multiple cell types [135]. Notably, the SIRT1 activator resveratrol has been shown to extend lifespan in various organisms, although whether sirtuin modulation extends lifespan in other organisms is controversial [136].

Recently, a link between epigenetic changes and the process of cellular senescence has emerged. In aged mouse stem cells, disruption of the Polycomb repressor complex 2 results in the de-repression of key senescent regulators including p16^ink4a^, leading to an induction of cellular senescence [59]. This is consistent with previous observations, reporting that downregulation of H3K27me3 (whose incorporation is performed by Polycomb repressor complexes) in aged pancreas correlates with deregulation of p16^ink4a^ [32,33]. Furthermore, epigenetic regulation has been implicated in age-associated increases in p16^ink4a^ in other stem cell compartments. Bmi1, a component of the Polycomb complex I that is required for the generation of self-renewing adult HSC and neural stem cells [137], can prevent premature senescence of neural stem cells by repressing p16^ink4a^ and ARF [138]. This is likely to act in concert with Hgma2, a transcriptional regulator highly expressed in young neural stem cells whose expression declines with age, as its genetic disruption leads to reduced self renewal throughout the central nervous system through increases in p16^ink4a^ and ARF [139]. These observations demonstrate that age-related epigenetic changes impact on regenerative capacity through regulating the process of cellular senescence (see Section 4).

These age-associated epigenetic modifications could underlie some of the transcriptional signatures associated with aging, such as the dysregulation of gero-miRNAs [140], a miRNA class which has been shown to affect longevity in *Caenorhabditis elegans* [141] and *Drosophila melanogaster* [142] as well as modulating stem cell behaviour in mammals. Interestingly, miRNAs regulate a wide variety of DNA damage response components (reviewed in Ugalde *et al.* [135]), and have been shown to play roles in other processes that affect regenerative capacity during aging such as cell metabolism and cellular senescence [143]. Furthermore, miRNA regulation is important for regeneration in species that do not exhibit an age-associated decline in this process such zebrafish [144,145] and salamanders [146,147]. Lastly, it is notable that some age-associated miRNAs can also regulate epigenetic modifiers, as is the case of miRNA-217, which can affect SIRT1 expression [148,149]. It is therefore likely that further research will uncover additional links between the age-related deregulation of miRNAs and the loss of regenerative abilities in various species.

### 3.5. Alterations in Proteostasis

The regenerative abilities of a cell depend on the maintenance of a functional proteome. Protein homeostasis, or proteostasis, is achieved by an interrelated network of pathways that collectively regulate and monitor protein production, localisation and folding, eliminate damaged, aggregated or misfolded proteins, and trigger cell cycle arrest or even cell death upon severe proteotoxic stress. Specialised proteins called chaperones control protein folding and localisation, while the autophagy and ubiquitin-mediated proteasome degradation systems mediate protein elimination. Defects in these important mechanisms are associated with increased cellular damage and protein dysfunction [145]. Critically, many studies have shown accumulation of misfolded and damaged proteins during aging, suggesting that the efficiency of the proteostasis machinery decreases during this process [150]. Indeed, age is the principal risk factor for most protein misfolding disorders [151]. In addition, most proteostasis mechanisms have been shown to decline with age in human and model organisms, including authopagy [152], the endoplasmic reticulum stress response [153], the ubiquitin-proteasome degradation system [154], and the mitochondrial unfolded protein stress response [64]. More recently, evidence has started to emerge supporting the importance of proteostasis for the maintenance of regenerative capacity. Studies in haematopoietic stem cells showed that deletion of the autophagy-related gene Atg7 leads to elevated ROS levels and HSC depletion [155,156]. Furthermore, inhibition of mTORC1, a potent regulator of autophagy, using rapamycin restores self-renewal and differentiation capacities in aged haematopoietic and intestinal stem cells. However, it is not yet clear whether mTORC1 inhibition exerts its anti-aging effects through the regulation of autophagy. In this connection, it is worth noting that mTOR activity is regulated by nutrient availability, and has also been implicated in the regulation of cellular senescence [157,158]. Indeed, rapamycin treatment can lead to considerable inhibition of cell senescence in various contexts [159,160]. It is therefore possible that mTOR could regulate regenerative capacity due to effects on nutrient sensing and/or cellular senescence, two emerging determinants of aging-related regenerative decline (see below). Nevertheless, a recent study has provided strong evidence supporting proteostasis dysregulation as a determinant of regenerative capacity declines with age [64]. Mohrin and co-workers found that the histone deacetylase sirtuin 7 (SIRT7) controls the mitochondrial unfolded protein response (mUPR) by regulating the expression of mitochondrial translation factors. By disrupting Sirt7 in mice, they showed that SIRT7 regulates the level of mUPR, proliferation, differentiation and reconstitution abilities in the haematopoietic stem cell compartment. Furthermore, they demonstrated that the levels of SIRT7 decrease with age in HSC and that their functional defects recapitulate those induced by SIRT7 elimination. Finally, they showed that they could rescue the defects in the aged HSC compartment by overexpressing SIRT7, suggesting that the age-dependent downregulation of this central mUPR component contributes to the age-dependent decline in HSC regenerative capacities.

### 3.6. Metabolic Changes

Mounting evidence suggests that metabolic pathways in various cell types, including stem and progenitor cells, change with age. In particular, signalling pathways involved in nutrient sensing such as the insulin-IGF-1, Akt-FOXO, AMPK and mTOR pathways, which link nutrient availability with other cellular functions, unsurprisingly play important roles in the maintenance of stem cell quiescence during aging [161]. These are also among the most evolutionarily conserved pathways involved in the control of aging at the organism level, from flies to humans [3]. In the case of regenerative cell types, age-related changes in these pathways can affect multiple cell functions. For example, deletion of the mTOR regulator TSC1, which enhances mTOR signalling, leads to loss of quiescence in HSC due to enhanced proliferation in the stem cell compartment [162]. Likewise, increased mTOR signalling leads to epidermal stem cell depletion through promotion of proliferation [163], which can be counteracted by treatment with the mTOR inhibitor rapamycin [159]. Moreover, inhibition of mTORC1 enhances HSC self-renewal during aging [162] and promotes maintenance of intestinal stem cells in mouse through non-autonomous effects on Paneth cells [164].

The importance of metabolic regulation as a contributing factor to age-related decreases in regenerative potential is further highlighted by the effect of caloric restriction on a number of regenerative systems. For example, multiple cycles of fasting have been shown to improve HSC self-renewal and regenerative capacities, in an IGF1-dependent manner [165], while short-term caloric restriction can revert age-associated increases in adipose-derived stem cells [166] and leads to enhancements in muscle stem cell function and repair [167]. Although the molecular mechanisms that mediate the beneficial effects of caloric restriction are not fully understood, it is clear that the IGF1 and mTOR networks play important roles [168].

It is possible that some of the metabolic changes that take place upon aging are a consequence of other age-related processes, such as telomere shortening and cellular senescence. Upon induction of senescence, cells undergo a series of phenotypic transformations that include metabolic reshaping. In the case of therapy-induced senescence, cells increase the rate of glucose metabolism and ATP production, likely as a result of proteotoxic stress [169]. Furthermore, cells undergoing telomere stress due to lack of telomerase exhibit a decrease in gluconeogenesis, impaired glucose tolerance and mitochondrial compromise, which are reversed by telomerase overexpression [13,170]. Thus, it is increasingly evident that metabolic changes can be brought about by many processes that are dysregulated during aging. Further research should clarify the level of metabolic changes and interconnectivity of metabolic changes with other factors that mediate age-associated declines in regenerative capacity.

### 3.7. Extrinsic Signals: Local, Systemic and Blood-Borne Factors

In addition to intrinsic changes in cells that participate directly in the regenerative response, changes in extrinsic factors such as the regeneration microenvironment, systemic molecules and the immune system, have also been associated with the decline in regenerative capacity during aging.

Age-related changes in the specialised microenvironment of stem cells, the stem cell niche, have been shown to affect the regenerative capacity of stem cells in various tissues, including muscle [171,172,173], intestine [174] and the central nervous system [175]. Among these, the best-characterised example is skeletal muscle. Muscle regeneration relies on an intricate network of interactions between the resident stem cells, the satellite cells, and the constituents of their niche, which include fibro-adipogenic progenitors (FAPS), immune cells and muscle fibres. Many molecular mediators of these interactions have been found to be dysregulated upon aging, leading to defects in muscle regeneration. Notably, the plasma membrane levels of Delta, a Notch-1 ligand, decrease in aged muscle fibres leading to defects in the activation of satellite cell priming and proliferation [173]. In agreement with this, forced activation of Notch signalling in aged mice muscle restores its regenerative potential following injury [173]. Together, this suggests that age-related alterations in Notch signalling in the satellite cell niche are responsible for the decrease in muscle regenerative potential [176]. Further investigations revealed that, in addition to deregulation of the Notch pathway, aged muscle fibres exhibit increased levels of FGF2 signalling, which drives satellite cells out of quiescence, promoting stem-cell depletion and loss of regenerative capacity [172]. Nevertheless, it has recently been proposed that the increase in FGF2 levels in aged muscle fibres constitutes a compensatory mechanism for the loss of FRFR1 activity in aged satellite cells, an intrinsic alteration that results in the constitutive activation of the p38 kinase leading to loss of asymmetric polarization/self-renewal in muscle stem cells [177]. In this connection, it is worth noting that p38 activation has also been linked to the promotion of cellular senescence, a process that is prevalent among aged muscle stem cells [59]. It is therefore possible that age-related p38 activation contributes to the decline in muscle regenerative abilities by both disrupting the asymmetric polarization of satellite cells as well as increasing their predisposition to undergo cellular senescence. Hence, these studies illustrate that both niche changes as well as cell intrinsic alterations are important determinants of the decline in regenerative abilities during aging.

Although rather understudied, another important element of the regenerative niche that can influence the outcome of regenerative processes in many contexts, including aging, is the regenerating nerve. A transient dependence on the concomitant regeneration of the nerves is seen in many regenerative processes, from tissue homeostasis to the regeneration of complex structures, in both vertebrate and invertebrate models [178]. The importance of the nerve as a critical determinant of regeneration is evident during salamander limb regeneration, where denervation of a regenerating limb curtails the proliferation of blastema cells and thus regrowth of the structure [178]. A similar requirement for nerve regeneration has been observed in zebrafish fin and barbels, starfish arms and annelid segments [178,179]. The presence of nerves is also critical in certain mammalian regeneration contexts, including bone marrow HSC, hair follicle and taste bud regeneration. In the case of the hair follicle, the nerves act as a source of Sonic Hedgehog that determines the ability of the upper bulge stem cells to become epidermal stem cells [180]. In the bone marrow, the association between HSC and Schwann cells has been shown to be a determinant of their quiescence [181]. More recently, it was demonstrated that innervation regulates myocyte proliferation and heart regeneration in neonatal mice as well as in zebrafish [182]. These are important considerations in the light of the general decline in nerve regeneration observed during aging [183]. Indeed, a decline in gustatory nerve regeneration is associated with the impairment in taste bud regeneration in aging mammals [27]. Together, these considerations raise the possibility that age-related changes in nerve regeneration capacity or functionality contribute to declines in regenerative processes in associated tissues.

The mechanical properties of the microenvironment are critical regulators of cell behaviour and are often subject to age-dependent changes. In various contexts, ageing leads to alterations in the composition of the extracellular matrix (ECM) which can affect stem/progenitor cell functions such as self-renewal, quiescence and differentiation [184]. In the case of muscle, ECM changes upon ageing lead to increases in myofibre stiffness [185], with consequences for satellite cell function. Changes in substrate elasticity regulate skeletal muscle stem cell self-renewal in culture [186]. Furthermore, recent studies of isolated muscle fibres suggest that the increase in stiffness in aged myofibres leads to an impairment in proliferative capacity of the associated satellite cells [187]. These observations highlight the relevance of the mechanical microenvironment as a factor that affects variations in regenerative capacity upon ageing.

Besides local cues, systemic factors play critical roles in regenerative processes and are subject to changes during aging. The importance of the systemic environment was initially highlighted by grafting experiments as well as heterochronic parabiosis. Grafting of old muscle into young rats was shown to improve the regenerative capacity of the grafted muscle, whereas grafting of young muscle into old hosts had the opposite effect, suggesting that age-related changes in the systemic environment impact on the ability to regenerate [188]. This notion was extended further by observations using heterochronic parabiosis, whereby the circulatory systems of two animals of different ages are conjoined. Heterochronic pairing of young and old mice results in improved regenerative capacities in skeletal and cardiac muscle, enhanced neurogenesis and remyelination in the old parabiont [176,189,190,191,192]. These observations prompted a search for the molecular factors that underlie this age-related systemic regulation of regenerative processes. To date, a handful of putative regulators exhibiting age-related alterations have been identified, including members of the Wnt [193] and TGF-β families (such as GDF11 [190]), cytokines such as CCL11 [192], and hormones such as oxytocin [194]. The role of hormones as systemic regulators of regeneration is elegantly exemplified by the phenomenon of deer antler regeneration, one of the fastest organogenesis processes in the natural world, which is linked to the annual breeding cycle and hence controlled by fluctuations in the concentration of sex hormones such as oestrogen [195,196] and liver-produced insulin growth factor [197]. Indeed, this unconventional mammalian model provided one of the earliest suggestions that life stage dependent fluctuations in systemic factors could act as regulators of regenerative abilities.

Interventions that counteract age-related alterations in the aforementioned factors including parabiosis, pathway inhibition/activation, or systemic supplementation, lead to improvements in the regenerative abilities of the target tissues. However, it is worth noting that these improvements are partial, as these interventions are unlikely to counteract cell-intrinsic changes that impair regenerative abilities during aging.

Among the systemic changes that take place during aging and could impact on regenerative abilities, age-related alterations in the immune system are currently attracting considerable interest. It is well established that highly regulated, transient immune responses play important roles in regeneration and tissue repair contexts, as demonstrated in various systems including the mammalian muscle [198,199], the zebrafish brain [200] and fin [201], and the axolotl limb [202]. The balance of pro-inflammatory *versus* anti-inflammatory components, in particular macrophages, can affect the regeneration outcome, with pro-inflammatory responses usually leading to tissue atrophy or fibrosis. This is highly relevant to mammalian aging, as this process is characterised by an increase in inflammatory responses [203] as well as a decrease in the functionality of the adaptive immune system [204]. Indeed, age-related changes in macrophages have been shown to contribute to the reduction in regenerative processes such as remyelination in the mammalian CNS [191]. Furthermore, an age-associated switch towards the production of pro-inflammatory cytokines accounts for the decline in epidermal regeneration in aged mammals. The threat posed by inflammatory responses during aging is further aggravated by the age-related accumulation of senescent cells (see discussion below), which can itself be promoted by chronic inflammation [205]. Senescent cells secrete a variety of pro-inflammatory cytokines and thus could form part of a positive feedback loop that hinders regenerative responses during aging. Furthermore, this accumulation of senescent cells is likely the result of failures in clearance mechanisms that depend on the immune system [206,207], which are thought to decline with age in mammals [208]. It is noteworthy that organisms such as salamanders, which do not exhibit an age-related decline in regenerative capacity, do not show age-related accumulation of senescent cells, and this is due to an effective mechanism of macrophage-dependent immunesurveillance that seems to be maintained throughout lifespan [209]. It would be of great interest to investigate the basis of this surveillance mechanism as well as the overall characteristics of the salamander immune response, as this could offer insights into the success and maintenance of regenerative capacities upon aging, as well as inspiring therapeutic strategies for improving immune-mediated targeting of senescent cells.

Lastly, recent research has uncovered another factor that is likely to impact on regenerative abilities during aging: the gut microbiota. In the past few years, there has been a major effort in defining the characteristics of the gut microbiota, its potential roles and its impact on its host. This has that revealed numerous aspects of an organism’s physiology are affected (at the local or systemic levels) by the composition of its gut microbiota, ranging from pathogen protection, metabolite synthesis, and detoxification, to the modulation of nervous, immunological and developmental processes [210,211]. Notably, the composition of the gut microbiota changes through the lifespan of individuals, and this can lead to changes in physiological processes regulated by the gut. Studies in the intestine of *D. melanogaster* have uncovered a role for the gut microbiota in the modulation of regenerative responses, by showing that gut bacteria promote intestinal epithelium renewal through the induction of intestinal stem cell proliferation [212]. This has also been suggested by recent studies in mice intestinal organoids [213]. Therefore, it is possible that age-related changes in the gut microbiota lead to defects in tissue homeostasis, repair and regeneration. In support of this idea, the studies in the fly intestine also revealed that age-related defects in intestinal epithelium renewal are reversed in the absence of indigenous bacteria [212]. Furthermore, it is worth noting that the gut flora can regulate other processes known to affect regenerative responses, including cell metabolism (through the regulation of insulin production), oxidative processes and immune system maturation and function. Although largely unexplored, the study of the impact of the gut microbiota on regenerative responses presents a promising area for future studies.

## 4. A Link between Cellular Senescence, Aging and Regenerative Capacity

In recent years, it has become clear that the process of cellular senescence underlies many aspects of aging [214], including decline in the capacity to sustain tissue homeostasis and regeneration. Due to its nature, it constitutes an important point of convergence of many factors that impair regenerative capacities including genomic instability, telomere attrition, epigenetic changes, oxidative damage, metabolic alterations, dysregulation of cellular communication and inflammation, representing an attractive target for therapeutic intervention.

Cellular senescence is traditionally understood as a state of permanent cell cycle arrest accompanied by various phenotypic alterations, which is induced by stimuli including exposure to DNA damage, telomere erosion, epigenetic alteration of senescence regulators, oxidative damage and oncogenic stress. Although the nature of the molecular events involved in the establishment of senescence is still under investigation, the p53/p21 and the p16^ink4a^/Rb pathways have been shown to play key roles in most senescent settings, chiefly through their effects on cell cycle inhibition for the promotion of growth arrest [215,216,217,218,219]. Like apoptosis, senescence functions to prevent the propagation of damaged cells, constituting a powerful mechanism of tumour suppression. However, in contrast to apoptotic cells, senescent cells remain metabolically active and undergo a series of phenotypic transformations which include the expansion of mitochondrial and lysosomal networks (leading to high levels of senescence-associated-β-galactosidase (SAβgal) activity, the best-known senescent cell marker [220], up-regulation of DNA checkpoint proteins such as p53, p21 and p16^ink4a^, rearrangements in nuclear architecture and heterochromatin organization, and the acquisition of a senescence-associated secretory phenotype (SASP) which comprises growth factors, proinflammatory cytokines, chemokines and matrix-remodelling proteins [221,222,223]. Despite their beneficial functions in tumour-suppression, senescent cells can have detrimental effects on biological processes. These effects can be cell intrinsic, as in the case of the direct abrogation of proliferative capacity or alterations in cell function, or cell extrinsic, such as the paracrine induction of senescence in neighbouring cells [224,225] and the promotion of inflammation [206], which are mediated by the SASP. In particular, these cell-extrinsic effects have been proposed to contribute to tissue degeneration, malfunction and decline in regenerative capacities.

The negative functions of cellular senescence are of particular relevance to aging, as senescent cells have been shown to accumulate in various tissues (including skin, lung, liver, spleen and kidney) in mammals as they age [226,227]. Critically, recent evidence in mouse models established a casual link between the accumulation of senescent cells and a number of age-related disorders. In a seminal study by Van Deursen and co-workers, genetic elimination of p16^ink4a+^ senescent cells in mice with a progeroid background caused by BubR1 deficiency was able to delay the onset of age-related disorders including cataracts, sarcopenia, osteoporosis and subcutaneous fat loss [228]. These results imply that cellular senescence is an important contributor to age-related decay. Furthermore, they suggest that removal of senescent cells could prevent or delay tissue dysfunction and extend healthspan [214,228].

**Figure 4:**
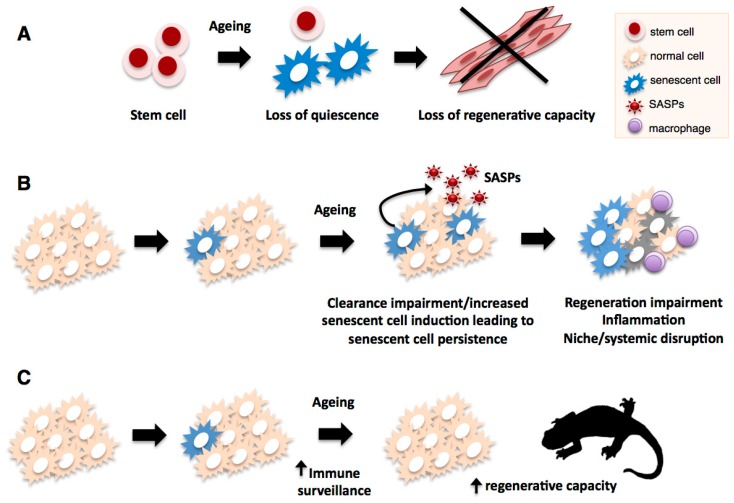
Impact of cellular senescence on regenerative processes during aging. Senescent cells accumulate in most organisms as they age, and this can negatively affect various regenerative processes. In the case of aged mammalian stem cells such as satellite cells (**A**), an age-associated switch to cellular senescence leads to loss of quiescence resulting in impaired regenerative ability; In addition to direct effects on regenerative capacity, senescent cells could promote tissue disruption, inflammation and niche or systemic alterations (**B**) through their phenotype (SASP), leading to further regenerative impairment; Senescent cell accumulation through aging can result from increases in stimuli that trigger cellular senescence (e.g., ROS, DNA damage, telomere attrition) or impairments in the immune-mediated clearance mechanism. In contrast to mammals, salamanders (**C**) have highly efficient mechanisms of senescence immunesurveillance which prevent senescent cell accumulation and could support their extensive regenerative abilities through lifespan.

With regards to cell types involved in regenerative processes, the accumulation of senescent progenitor (*i.e*., endothelial progenitor cells) or stem cells (*i.e*., HSC, satellite cells, NSC) has been observed *in vivo* during mammalian aging and in progeroid syndrome mouse models. As so far there is no unambiguous senescence marker available, all observations are based on the combined detection of various senescent cell markers, such as SAβgal, lack of proliferation markers,γ H2AX foci and p16^ink4a^ protein levels [229]. Of these, many studies have focused on p16^ink4a^ expression, as it is both a marker (found up-regulated in many senescence contexts) as well as an established inducer of senescence. Its overexpression has been shown to contribute to the replicative failure of many regenerative cell types [69,70], while its downregulation or genetic deletion has been shown to ameliorate the age-associated functional and proliferative impairment in stem and progenitor cells [68,230]. This led to the suggestion that cellular senescence could be an important factor underlying the decline in regenerative capacity with aging. In support of this idea, modifications that lead to cellular senescence, such as telomerase inhibition and nucleotide-analogue induced replicative damage, result in reduced tissue repair and regenerative capacities [231]. Furthermore, the aforementioned studies in the BubR1 mice hinted that selective elimination of p16^ink4a+^ senescent cells could be beneficial for regenerative processes, given the improvements observed in epithelium structure and sarcopenia [228]. More recently, direct evidence of the role of senescence as a key mediator of age-related decline in regenerative capacities came from studies in mouse muscle regeneration, which revealed that geriatric muscle stem cells loose their reversible quiescent state during aging by undergoing cellular senescence (Figure 4) [59,232]. This switch, which renders cells unable to activate and expand upon muscle injury, is brought about by an age-related increase in p38 pathway activity and the loss of polycomb repressive complex activity, which result in increased p16^ink4a^ expression and senescence induction. Importantly, pharmacological inhibition of p38 or specific silencing of p16^ink4a^ in geriatric satellite cells restores both their reversible quiescence and regenerative functions [59,232]. These studies suggest that maintenance of the quiescent state relies on the active repression of senescence pathways, and that cellular senescence is a major contributor to the age-related decline in regenerative abilities.

Besides the direct impact of cellular senescence on stem cell quiescence, this process could affect regenerative capacities indirectly (Figure 4), through the promotion of tissue degeneration, malfunction and inflammation, brought about by the senescence-associated phenotype (SASP). These processes could lead to niche and/or systemic alterations in the regenerative environment that result in impairments in regenerative abilities. In addition, it is possible that particular components of the SASP could impact on the regenerative cell types directly and affect their regenerative potential or function.

The association of cellular senescence with negative outcomes at the organism level has recently revived discussions on its evolutionary rationale. Even though cellular senescence has tumour-suppressor functions that could explain its persistence, so does apoptosis. Moreover, apoptosis could be considered a more effective mechanism, as it involves direct elimination of the target cells. It is possible that the negative effects of cellular senescence are manifested mainly during the post-reproductive period, and hence escape the challenges of selection. Alternatively, it is conceivable that senescent cells play positive roles in physiological contexts. In support of this view, the transient induction senescent cells have recently been found to contribute to tissue remodelling during mouse embryonic development [233,234], wound healing [235,236], and fibrosis restriction in organs such as liver [237] and heart [238]. Though distinct, these processes are each relevant to tissue regeneration. Therefore, it is possible that cellular senescence, when induced transiently, could play positive roles in regenerative responses. Although this suggestion has not yet been addressed directly, studies in the salamander limb regeneration model have recently offered significant insights. These investigations revealed that senescent cells are recurrently induced during the intermediate stages of regeneration, coinciding with the period of generation/expansion of regenerative progenitors, and are subsequently eliminated by a highly effective mechanism of senescence immunesurveillance dependent on macrophages [209]. Interestingly, senescent cells are not induced during normal limb development in this model, indicating that this phenomenon is specific to regeneration. The implications of these findings are varied. First, they constitute the first report of senescent cell induction during regeneration, and open the door to analysing whether transient induction of cellular senescence contributes to regeneration, an important future direction for both the Aging and the Regeneration fields. This has significant therapeutic implications for the design of anti-aging or regenerative strategies that target senescent cells: were they to play a positive role at certain stages of regeneration, their targeting should be avoided during that period. Second, they uncovered a new, highly efficient mechanism of immune surveillance that operates in normal and regenerating tissues, which correlates with a lack of age-related accumulation of senescent cell in salamanders (Figure 4). This mechanism has been proposed to support the ability of this organism to undergo regeneration throughout its lifespan [209]. Its study could deliver new approaches for the elimination of senescent cells to promote regenerative processes and healthspan improvement in organisms such as humans.

## 5. Avenues for Therapeutic Interventions

The advances in our understanding of the factors that modulate the decline in regenerative abilities have pinpointed areas of potential clinical relevance. At present, therapeutic strategies that counteract defects in regenerative capacities are somewhat lacking. In any case, in designing such strategies, the type of regeneration to be improved (whether it is tissue turnover or structural replacement), the defects to be overcome, the cell types to be regenerated and the characteristics of the regeneration niche should be considered.

**Figure 5:**
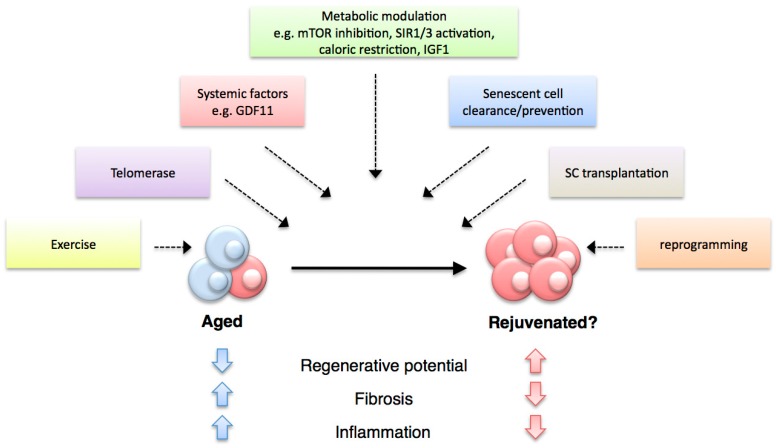
Therapeutic strategies for improving regenerative capacity.

Although it could be argued that transplantation of stem cells could constitute a viable and clinically relevant option in various contexts, it faces many challenges such as the acquisition and survival of sufficient quantities of cells for the process, immunocompatibility and cancer risk issues, and the fact that transplantation of young cells may not be able to overcome extrinsic influences that could underlie the decay in regenerative abilities in an aged host. Hence, addressing the particular alterations in the processes that drive age-related regenerative decays could represent a more fruitful alternative. This would involve the specific targeting of extrinsic and intrinsic factors (and, most likely, a combination of them), taking into account tissue/system/timing specific requirements, and balancing the benefits for the organism against secondary outcomes associated to each manipulation. Various approaches have emerged from studies in experimental models (Figure 5), however they differ in their capacity to improve regeneration, the secondary effects associated to them and thus their therapeutic promise. For example, even though the manipulation of certain pathways involved in the maintenance of genome stability (*i.e*., p53, p16^ink4a^) and telomerase reactivation represent powerful strategies for counteracting the loss of regenerative abilities upon aging, the associated risk of tumourigenesis represents a big caveat to their application. In contrast, systemic approaches such as caloric restriction, excersise and controlled modulation of key metabolic pathways such as mTOR, Igf1 and sirtuins hold better therapeutic prospects. In addition, much emphasis has recently been put on the use of systemic factors for the promotion of regeneration (e.g., GDF11). Although these certainly have good potential, further studies are required in order to start considering their application in a clinical setting.

In light of recent discoveries, a strategy that has aroused considerable interest within the Aging field is the selective elimination of senescent cells [214]. Studies in mouse models have shown its potential as a means to enhance regenerative abilities [59,232] as well as to improve a number of age-associated pathologies [228]. However, senescent cell clearance has so far been achieved, in most cases, through genetic ablation of senescent cells based on p16^ink4a^ expression. Although p38 inhibition in pre-senescent cells could constitute an alternative [232], p38 functions are so ubiquitous that it is unlikely that p38 inhibitors could be broadly used, unless they are applied to isolated regenerative sub-types. These considerations have led to the search for “senolitics”, molecules that selectively promote the elimination of senescent cells. Progress has been made with the recent finding that a combination of such compounds, dasatinib and quercetin, leads to improved senescent cell elimination in a mouse model [239]. However, its effects on regenerative abilities (and many other physiological processes) were not evaluated in this study and remain to be tested. Alternatively, it is possible that senescent cell elimination can be achieved by the promotion of their natural mechanism of destruction, which consists of elimination through immunesurveillance [206]. Although there are at present no means to achieve this, the recent finding of a highly efficient immune mechanism of senescent cell clearance in the archetypical regeneration model, the salamander [209], has opened up new possibilities for exploring this alternative.

Multiple clinical approaches, targeting some of the key factors that affect regenerative capacity upon aging, have been proposed for the improvement of regenerative processes in mammals. These are based on the elimination of damaged cells (e.g., senescent cell clearance) or the improvement of regeneration potential in regenerative cell types (e.g., epigenetic reprogramming). They may also result in decreased inflammation and reduced fibrosis during tissue repair. Therapeutic applications are likely to involve combinatory approaches, counteracting the effect of both cell-intrinsic as well as cell-extrinsic factors.

The lack of information on the physiological consequences of certain manipulations, though attractive, represents a barrier to their implementation. This includes the reversion of epigenetic changes through induced reprogramming to pluripotency. This strategy has been successfully used to reprogramme aged haematopoietic progenitors [240] and T cells [241,242], and the resulting iPSC were differentiated following expansion into the respective cell types, leading to “rejuvenated” haematopoietic or lymphocyte populations. However, it is unclear what the long-term consequences of these plasticity changes are, whether they cause any impairment in cellular function or even increase cancer propensity. Likewise, it is not clear if reprogrammed cells are indeed rejuvenated [243]. These issues should be addressed by future research in order to unlock the potential of reprogramming as a therapeutic strategy for improving regenerative processes.

These observations have raised questions such as what is the nature of rejuvenation in molecular terms, and whether any of the modifications discussed herein can actually induce cells to revert to a more youthful status [244]. These issues are also pertinent in the context of super-regenerator organisms such as salamanders, which can regenerate complex body parts multiple times throughout their lives, without any decay in regenerative abilities. Do they exhibit mechanisms to induce reversion to a more youthful cellular state? This question needs to be considered in the light of the regenerative strategy used by these organisms, as it differs from the unidirectional stem cell-based regeneration seen in mammals. In salamanders, the main regenerative strategy is partial dedifferentiation (a type of limited reprogramming) of adult cell types followed by extensive proliferation and redifferentiation [48,245]. This process involves the reversion of certain epigenetic modifications [246,247], but whether dedifferentiation is able to revert aged adult tissues to a youthful status is unknown. In this connection, it is worth noting that newt lens regeneration, an age-resistant process, takes place by transdifferentiation of somatic cells [51]. Interestingly, it has been observed that this process involves a step of dedifferentiation and expression of pluripotency genes such as Sox2, c-myc and Klf4 [248], and this could represent a reversion of age status. Certainly, it is possible that the nature of the regeneration strategy, combined with effective mechanisms to overcome processes that negatively impact on regeneration in other systems, such as cellular senescence [209] and possibly others, could explain this remarkable ability to withstand aging and age-related declines in regenerative capacity. This a possibility that should be addressed by further studies, as it has significant implications of fundamental and therapeutic nature.

In this context, it is also interesting to consider other extreme regenerators such planarian flatworms, which are able to achieve whole body regeneration through a stem cell based mechanism [38,249]. In contrast to mammalian stem cells, a single planarian neoblast (the planarian stem cell) has the potential to reconstitute the entirety of planarian tissue types [250]. This raises further questions, such as what underlies the extreme potency of planarian stem cells, how this is related to their apparent immortality, and whether they share any strategies for the preservation of cellular and genomic integrity with germ cells. Together, these considerations illustrate that much is yet to be learned from these powerful regeneration models regarding how to promote effective regeneration throughout lifespan. Undoubtedly, this constitutes a promising area for future studies.

## 6. Conclusions

The ability to regenerate tissues and organs is widespread across the animal kingdom. However, there are marked changes in regenerative capacity through phylogeny, ontogeny and aging. Whilst a number of organisms exhibit extensive regenerative faculties which do not decline through their lives, a progressive decay in the capacity to undergo regenerative processes is observed as most organisms age. A considerable number of factors contribute to this decline in regenerative ability. Their extensive overlap with the recently defined “Hallmarks of Aging” highlights the interrelated nature of the dwindling of regenerative capacities and the process of aging. Establishing the relative importance of such factors, the extent of their effects on different regeneration systems, the degree of interconnectivity between them and the points of convergence will be crucial for understanding the nature of age-related regenerative declines and designing fruitful therapeutic strategies to overcome them. It is becoming increasingly clear that certain factors, such as cellular senescence, constitute common denominators which impact on various regenerative systems and thus hold great promise for clinical intervention. However, despite the progress made so far, it is also evident that we are far from reaching a full understanding of the interplay between regenerative capacity and aging. Further research will benefit from studies in both vertebrate and invertebrate models of age-related regenerative decline, as well as from work in organisms where these capacities are not affected by aging, such as salamanders. Together, these approaches will deliver important insights into the variations of regenerative capacity through lifespan.
